# Co-Production of an Online Medical Student Conference: Inspiring Interest in Psychiatry

**DOI:** 10.1192/bjo.2022.155

**Published:** 2022-06-20

**Authors:** Georgina Edgerley Harris, Sonya Rudra, Rachel Swain, Abigail Swerdlow

**Affiliations:** 1 South West London and St George's Mental Health NHS Trust, London, United Kingdom; 2 East London NHS Trust, London, United Kingdom; 3 Central & North West London NHS Foundation Trust, London, United Kingdom

## Abstract

**Aims:**

The aim of this project was to create a Pan-London event to increase awareness and enthusiasm of medical students for Psychiatry as a specialty. In addition to a longer term goal of ultimately increasing recruitment to the specialty once students qualify, this event aimed to bring Mental Health to the forefront of the minds of future doctors.

**Methods:**

Psychiatry Teaching Fellows from different trusts created a virtual educational event targeted at medical students in all years across London universities. It was co-produced with the student Psychiatry Societies across the London Universities. This encouraged student engagement from the ground level and fostered an environment of collaboration between students and Doctors. The event was free to attend and was supported by the Royal College of Psychiatry, London Division. The conference programme showcased the various facets Psychiatry has to offer from a global perspective, including Women's Mental Health, Forensic Psychiatry, research and volunteering around the world.

**Results:**

The conference welcomed 263 attendees. 92 of the attendees completed a feedback questionnaire at the end of the session. The majority of respondents were from London universities and fairly evenly distributed amongst medical school year groups. 99% of those completing the questionnaire found the session interesting (scoring 3 or more out of 5 on a 5 point Likert scale). 98% of respondents reported that they found the session widened their view of Psychiatry. 78% were already considering a career in Psychiatry. 96% felt more likely to pursue a career in Psychiatry following the conference (scoring 3 or more out of 5 on a 5 point Likert scale). Open-text feedback indicated that attendees had found the sessions interesting and particularly valued the range of topics.

**Conclusion:**

Extra-curricular events are a fantastic chance to broaden medical students’ views of the specialty of Psychiatry. A virtual platform creates opportunities for audiences to hear from a vast array of expert speakers, which might not otherwise be possible in person, and creates a community of like-minded students in a safe environment. Whether or not students go on to pursue the field themselves later on in their training, events such as this bring awareness of Psychiatry and its impacts to the foreground. It is hoped that, in future, further co-produced events between the Royal College of Psychiatry and university Psychiatry societies, can continue to inspire medical students.

